# Alterations of Gut Microbiome and Metabolite Profiles Associated With Anabatic Lipid Dysmetabolism in Thyroid Cancer

**DOI:** 10.3389/fendo.2022.893164

**Published:** 2022-06-03

**Authors:** Ganghua Lu, Xiaqing Yu, Wen Jiang, Qiong Luo, Junyu Tong, Suyun Fan, Li Chai, Dingwei Gao, Tingting Qiao, Ru Wang, Chengwen Deng, Zhongwei Lv, Dan Li

**Affiliations:** ^1^Department of Nuclear Medicine, Shanghai Tenth People’s Hospital, School of Medicine, Tongji University, Shanghai, China; ^2^Clinical Nuclear Medicine Center, Tongji University School of Medicine, Shanghai, China; ^3^Imaging Clinical Medical Center, Tongji University School of Medicine, Shanghai, China; ^4^Institute of Nuclear Medicine, Tongji University School of Medicine, Shanghai, China

**Keywords:** differentiated thyroid cancer, gut microbiota, metabolomics, lipid metabolism, diet

## Abstract

**Background:**

Currently, the high morbidity of individuals with thyroid cancer (TC) is an increasing health care burden worldwide. The aim of our study was to investigate the relationship among the gut microbiota community, metabolites, and the development of differentiated thyroid cancer.

**Methods:**

16S rRNA gene sequencing and an integrated LC–MS-based metabolomics approach were performed to obtain the components and characteristics of fecal microbiota and metabolites from 50 patients with TC and 58 healthy controls (HCs).

**Results:**

The diversity and richness of the gut microbiota in the TC patients were markedly decreased. The composition of the gut microbiota was significantly altered, and the *Bacteroides* enterotype was the dominant enterotype in TC patients. Additionally, the diagnostic validity of the combined model (three genera and eight metabolites) and the metabolite model (six metabolites) were markedly higher than that of the microbial model (seven genera) for distinguishing TC patients from HCs. LEfSe analysis demonstrated that genera (*g_Christensenellaceae_R-7_group, g_Eubacterium_coprostanoligenes_group*) and metabolites [27-hydroxycholesterol (27HC), cholesterol] closely related to lipid metabolism were greatly reduced in the TC group. In addition, a clinical serum indicator (total cholesterol) and metabolites (27HC and cholesterol) had the strongest influence on the sample distribution. Furthermore, functional pathways related to steroid biosynthesis and lipid digestion were inhibited in the TC group. In the microbiota-metabolite network, 27HC was significantly related to metabolism-related microorganisms (*g_Christensenellaceae_R-7_group*).

**Conclusions:**

Our research explored the characteristics of the gut microecology of patients with TC. The findings of this study will help to discover risk factors that affect the occurrence and development of TC in the intestinal microecology.

## Introduction

Thyroid cancer (TC) is one of the most common malignant tumors of the head and neck. The incidence rate of TC has gradually increased worldwide in the past several decades, which is potentially caused by excessive exposure of individuals to disease risk factors (1). Thus far, numerous studies have focused on diet and metabolism, especially obesity, as potential important risk factors for increased morbidity in individuals with TC (2–4). Previous studies have shown that as the body mass index (BMI) increases, the risk of thyroid cancer is subsequently greatly increased (3). Moreover, obesity is an independent risk factor for thyroid malignant tumors, and this association may be further aggravated by metabolic abnormalities (5). In fact, diet plays a decisive role in the human intestinal microecological environment and can, thereby, influence physiological mechanisms of the human body. These findings provide a novel perspective for the association between intestinal flora and thyroid cancer (6).

In recent years, multiple studies have collected small fecal samples to demonstrate that the composition of the gut microbiota in patients with TC is markedly altered compared with healthy controls (HCs). These studies further established an intestinal flora model and metabolite model to characterize thyroid cancer (7–9). Our previous study increased the sample size to verify the connection between TC and gut microbiota and predict a more convincing model of microbial diagnosis and functional pathways (10).

Although these studies have already revealed the underlying associations between the intestinal flora and metabolites in thyroid cancer, the synergistic mediation effects of the gut microbiota and its corresponding metabolites on lipid metabolism disorders in individuals with thyroid cancer were not thoroughly screened. The fecal flora has expansive interrelationships, and the elements that determine the overall microecological balance hinge on the importance instead of the abundance of certain species in the gut network system (11). However, it is still not completely clear which fecal genera and metabolites are of relative importance for thyroid cancer. Therefore, it is urgent to understand if or how lipid metabolism disorders induce thyroid carcinoma by establishing an internetwork model of microbiota and metabolites. This type of network model could not only characterize the crucial and central factors but also reveal potential relationships in multiomics analyses (12).

According to the evidence above, the aim of our study was to investigate the essential fecal genera and metabolites and validate the coexistence relationship between intestinal microbiota and metabolites in patients with TC and in HCs using 16S rRNA sequencing and liquid chromatography mass spectrometry analysis.

## Materials and Methods

### Study Subjects and Design

This study recruited 50 TC patients (22 males, 28 females, ages 16-69 years, median age 41) from the Department of Nuclear Medicine, Shanghai Tenth People’s Hospital between May 2017 and February 2019. The control group consisted of 58 healthy volunteers (22 males, 36 females, ages 22-71 years, median age 44) who visited this hospital for their routine physical examination. The diagnosis of TC was based on the following criteria: (i) clinically diagnosed with differentiated thyroid cancer within six months (13), (ii) age between 18 and 75 years, female (nonpregnant) or male without history of other malignancies, and (iii) receiving surgical treatment. The healthy volunteers were age-, sex- and BMI-matched with the TC patients. The following patients were also excluded: (i) patients confirmed not to have thyroid cancer on postoperative pathology, (ii) subjects suffering from a gastrointestinal disease or any other severe physical or mental disease, and (iii) subjects with a history of probiotics, orally ingested antibiotics, or any other similar drug within two months before fecal sampling. Fecal samples of the TC patients were collected prior to surgery. The following indices were obtained from the eight-hour fasting blood samples: (i) thyroid function indices, including serum free triiodothyronine (fT3), serum free thyroxine (fT4) and serum thyroid stimulating hormone (TSH); (ii) thyroid antibodies, including serum anti-thyroid peroxidase antibody (TPOAb) and serum anti-thyroid autoantibodies (TgAb); and (iii) serum biochemical indicators, including alanine transaminase (ALT), aspartate transaminase (AST), triglycerides (TGs), serum total cholesterol (TCHO), high-density lipoprotein cholesterol (HDL), and low-density lipoprotein cholesterol (LDL). The diagnosis of TC was determined by two independent pathologists in Shanghai Tenth People’s Hospital. All research subjects were of Han nationality and resided in the eastern and southeastern provinces of China. This study was conducted in accordance with the principles of the World Medical Association and the Declaration of Helsinki and was approved by the Shanghai Tenth People’s Hospital Ethics Committee (No. SHSY-IEC-KY-4.0/16-18/01). Written informed consent was obtained from the subjects.

### Fecal Sample Collection, DNA Extraction and Sequencing

Fecal samples were collected the morning after an overnight fast (more than 8 hours). Fecal samples were snap-frozen with liquid nitrogen following collection and stored at -80°C (Haier, DW-86L626, China) (14). Microbial genomic DNA was extracted using the E.Z.N.A. soil DNA Kit (Omega Bio-Tek, Norcross, GA, USA) in accordance with the manufacturer’s protocol. A Nanodrop 2000 (Thermo Scientific, USA) was used to measure the DNA concentration. Extracted DNA was stored at -80°C until experiments were conducted. The primers (338-F: 5’-ACTCCTACGGGAGGCAGCAG-3’) and (806-R: 5’-GGACTACHVGGGTWTCTAAT-3’) targeting the V3-V4 hypervariable region of the 16S rRNA were used to amplify the extracted DNA samples by the polymerase chain reaction system (ABI GeneAmp 9700, USA). The amplicons were subsequently purified by AxyPrep DNA Gel (Axygen, CA, USA) and quantified by QuantiFluor-ST (Promega, USA). Sequencing was performed on an Illumina MiSeq platform (Illumina, San Diego, CA) to combine the purified amplicons at equimolar concentrations and perform end pair sequencing analysis.

### Fecal Metabolism

Fecal sample (50 mg) was weighted to an EP tube, and 400 µL extract solution (methanol: water = 4: 1) was added. Then they were homogenized by a Wonbio-96c high-throughput tissue crusher (Shanghai Wanbo Biotechnology Co., Ltd.) for 6 min at 50 Hz. Then, they were vortexed for half a minute and ultrasonicated for 30 min at 40 kHz (incubated in ice water). The mixture was incubated for 30 minutes at -20°C to precipitate proteins and then centrifuged at 13,000 g for 15 min at 4°C. The supernatant of the samples was transferred to fresh EP tubes and analyzed by ultra-performance liquid chromatography (UPLC) system coupled to a quadrupole-time-of-flight mass spectrometer (Triple TOFTM5600+, AB Sciex, USA) equipped with a electrospray ionization (ESI) source operating in both positive mode and negative ion mode. Additionally, 10 μL of supernatant from each sample was combined and mixed to serve as the quality control (QC) sample, which was injected randomly (every 8 samples) to screen the consistency of the analysis.

### LC–MS/MS Analysis as Well as Annotation

Nontargeted liquid chromatography-mass spectrometry (LC–MS/MS) analyses were performed for peak detection and comparison by importing the raw data into the Progenesis QI 2.3 software program (Nonlinear Dynamics, Waters, USA), and a data matrix composed of retention time (RT), peak intensity and mass-to-charge ratio (m/z) values was obtained. Additionally, qualitative and quantitative information for specific metabolites was acquired. Preprocessing was necessary before the multivariate statistical analysis, and the methods were as follows: (i) data were removed if missing values were more than 50% in the sample; (ii) based on one-half of the minimum metabolite value, the missing values were filled and the total peaks were normalized; and (iii) QC samples were eliminated if the relative standard deviation (RSD) was more than 30%. Finally, logarithmic transformation was performed for preprocessed datasets prior to further analysis. Principal component analysis (PCA) was used to analyze the cation and anion set data for QC analysis. Mass spectra of these metabolic features were identified by using the accurate mass, MS/MS fragments spectra and isotope ratio difference with searching in reliable biochemical databases as Human Metabolome Database (HMDB) and Kyoto Encyclopedia of Genes and Genomes (KEGG). Specifically, the mass tolerance between the measured m/z values and the exact mass of the target components was ± 5 ppm. For MS/MS-confirmed metabolites, only metabolites with MS/MS fragments scores above 30 were considered reliably identified. Otherwise, the metabolites had only tentative assignments.

### Microbial Analysis of 16S rRNA Gene Sequencing

The analysis of 16S rRNA gene sequencing data was performed in the Majorbio Cloud Platform (https://cloud.majorbio.com/). Paired-end reads were first assembled using FLASH (v1.2.11) (15). Then, the sequences were optimized by filtering and quality control using Fastp (v0.19.6) (16). We then used UPARSE (v 7.0.1090) to cluster sequences by a similarity of 97% into operational taxonomic units (OTUs) (17).

The annotation of each taxonomy was performed by the RDP Classifier (v2.11) and the Silva database (v132). To decrease the effect of spurious sequences, sequences of OTUs <0.005% were removed (18).

### Statistical Analyses

The alpha diversity was analyzed to determine the richness of species at the OTU level based on the Ace index. The beta diversity was analyzed using the unweighted UniFrac algorithm to compare the composition of bacterial communities among samples. On the Majorbio Cloud Platform, the analysis of principal coordinates and similarities were performed to determine whether the intergroup differences were significantly greater than the intragroup differences or not. The Partitioning Around Medoids algorithm was used to cluster individuals into two enterotypes. The differences at the phylum or genus levels between the two groups were assessed by the Kruskal–Wallis H test. Linear discriminant analysis effect size (LEfSe) analysis was applied to determine the significantly different genera between the TC and HC groups (19).

Novel microbial and metabolic predictors were explored using logistic regression by forward stepwise (likelihood ratio) methods (SPSS Statistics v22), lasso regression and random forest model analysis (R environment v.4.1.0) (19, 20). The diagnostic ability of the selected substances was characterized by receiver operator characteristic (ROC) curve analysis with comparisons of the areas under the curve (AUCs). Differences between two AUCs were evaluated by the *Z* test. The final predictive models were validated in an additional cohort, consisting of 20 patients with thyroid cancer and 20 healthy controls *via* measuring microbiota and metabolites. Canonical correspondence analysis (CCA) was performed to conduct correlation analysis of clinical and metabolic factors among samples. The Spearman correlation between the two groups was determined for each factor.

Differential metabolites between the two groups were screened by orthogonal partial least squares discrimination analysis (OPLS-DA) and Student *t*-test (data conformed to a normal distribution and satisfied the homogeneity of variance) in the SIMICA software program (v 16.0.2) (21). The analysis of functional pathways was performed in the Majorbio cloud and MetaboAnalyst platform (v5.0) (22). The network between changed genera and lipid metabolism was visualized by Cytoscape (v3.6) software (23). A difference of statistical significance was considered only when the value of *p* was less than 0.05.

## Results

### Demographics and Clinical Characteristics

Our research enrolled 50 patients with TC and 58 healthy volunteers. There were no significant differences in age and sex between the two groups. The baseline characteristics of the TC patients and HCs are summarized in **Table 1**. Then we analyzed fecal samples from TC and HC groups using 16S rRNA gene sequencing and untargeted metabonomic analyses of their gut microbiota and metabolites.

**Table 1:** Baseline characteristics of TC patients and HCs.

Variables	TC (n = 50)	HC (n = 58)	*P* value^†^
Age	42.54 ± 14.38	42.40 ± 13.80	0.958
BMI	22.31 ± 1.44	21.99 ± 1.38	0.236
fT3	4.71 ± 0.84	4.53 ± 0.71	0.233
fT4	12.96 (11.89-15.93)	12.58 (11.53-14.92)	0.332
TSH	2.1 (1.60-2.65)	2.34 (1.57-2.83)	0.671
TgAb	10.00 (10.00-10.25)	10 (10-10.25)	0.987
TPOAb	2.00 (2.00-2.00)	2 (2-2.18)	0.494
AKP	69.5 (52.93-80.25)	56 (49.95-72.85)	0.034
DBil	2.50 (2.08-2.92)	2.5 (2.1-3.02)	0.76
TBil	10.16 ± 3.37	10.34 ± 3.09	0.775
ALT	27 (20.9-35)	23.05 (16-31.45)	0.118
AST	28.85 (23.78-32.62)	27 (21.7-30.2)	0.107
GHb	5.58 ± 0.41	5.46 ± 0.36	0.108
TCHO	3.55 (4.38-4.82)	3.93 (4.20-4.75)	0.744
TG	1.16 (0.94-1.47)	1.25 (1.05-1.48)	0.293
HDL	1.48 (1.32-1.58)	1.48 (1.27-1.58)	0.820
LDL	2.53 (2.01-3.04)	2.60 (2.12-3.04)	0.626

^†^Comparisons between TC patients and HCs.

TC, thyroid cancer; HC, healthy control; BMI, body mass index; fT3, free triiodothyronine; fT4, free thyroxine; TSH, thyroid-stimulating hormone; TGAb, thyroglobulin antibody; TPOAb, thyroid peroxidase antibody; AKP, alkaline phosphatase; DBil, direct bilirubin; TBil, total bilirubin; ALT, alanine aminotransferase; AST, aspartate aminotransferase; GHb, glycated hemoglobin; TCHO, total cholesterol; TG, triglycerides; HDL, high density lipoprotein; LDL, low density lipoprotein.

### The Change of Diversity, Richness, and Microbial Composition of Intestinal Flora in the TC Group Compared With HCs

**Figure 1:**
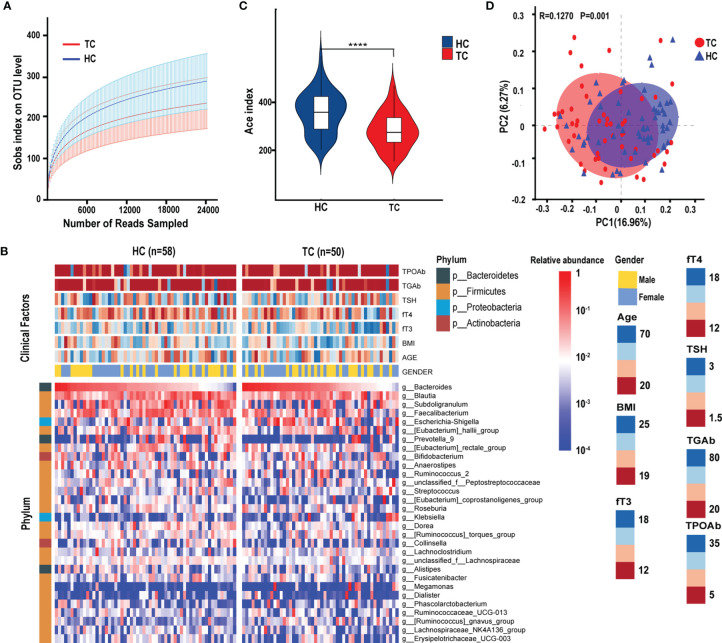
The diversity of gut microbiota in patients with TC (n = 50) and HC (n = 58) group. **(A)** The rarefaction curves between the number of read samples and the number of OTUs in Sobs index. **(B)** Annotated heatmap of clinical factors and gut microbial features of the top 30 genera based on Spearman distance and Ward hierarchical clustering. **(C)** The α-diversity of the bacterial communities based on the Ace index on the OTU level in TC and HC groups. **(D)** The PCoA based on unweighted UniFrac matrix on OTUs distribution showed that the composition of gut microbiota was significantly different between TC patients and HCs. ****p < 0.0001. TC, thyroid cancer; HC, healthy control; OTUs, operational taxonomic units; PCoA, principal coordinate analysis; BMI, body mass index; fT3, free triiodothyronine; fT4, free thyroxine; TSH, thyroid-stimulating hormone; TGAb, thyroglobulin antibody; TPOAb, thyroid peroxidase antibody.

After quality filtering, a total of 5,666,615 sequences from 108 samples were distributed into 1695 OTUs. According to the rank-abundance curves at the OTU level, the Good’s coverage indices of both TC and HC groups were higher than 99.5%, suggesting a satisfactory sequencing depth of the intestinal flora (**Figure 1A**). The microbial diversity characteristics and their relationship with clinical parameters are shown in **Figure 1B**. The top four phyla were *Firmicutes*, *Bacteroidetes*, *Actinobacteria*, and *Proteobacteria* (**Figure 1B**). At the genus level, *Bacteroides* was the most abundant genus, with an average relative abundance of 10.5%. Meanwhile, *Prevotella_9* and *Megamonas* showed bias distribution in our samples. We found that the enrichment of *Prevotella_9* (sequences in OTUs > 100) was lower in TC patients (n = 10, 20.0%) than in HCs (n = 26, 44.8%; *χ²* = 7.448*, p* < 0.05). Conversely, the enrichment of *Megamonas* was higher in TCs (n = 9, 18.0%) than in HC patients (n = 3, 5.2%; *χ²* = 4.474, *p* < 0.05) (**Figure 1B**). The α-diversity of gut microbes in TC patients was significantly decreased compared with that in HCs (*p* < 0.0001), which suggests that the richness of the gut microbiota is reduced in TCs (**Figure 1C**). The β-diversity was shown on the overall microbial composition of the microbiota based on the results of principal coordinate analysis (PCoA), which revealed obvious differences between the TC patients and HCs (*R* = 0.1270, *p* = 0.001, **Figure 1D**).

### Gut Microbiota Enterotypes Analysis

**Figure 2:**
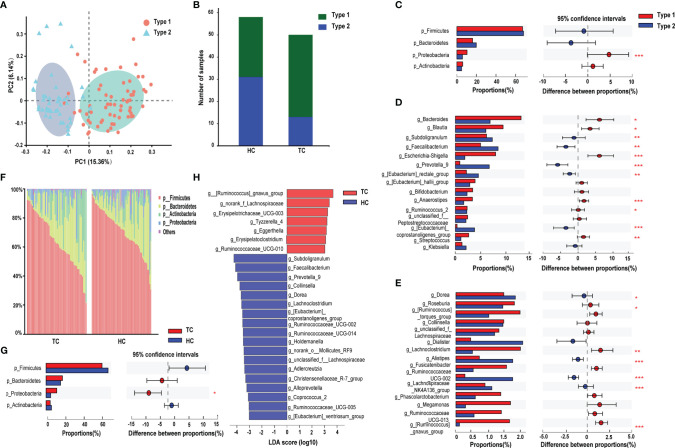
Enterotype analysis and alterations of gut microbiota composition in TC. **(A)** The PCoA based on unweighted UniFrac matrix on OTUs distribution showed that the composition of gut microbiota was significantly different between type 1 and type 2. **(B)** The proportion of the two enterotypes in TC and HC groups. The relative proportion and its difference of **(C)** the top 4 phyla and **(D-E)** top 30 genera between two enterotypes. **(F)** Relative proportion of bacteria on phylum level in TC patients and HCs. **(G)** Difference in the microbial proportion of the top 4 phyla between TC patients and HCs. **(H)** LEfSe analysis showed that the relative abundance of 25 genera was significantly different between TC patients and HCs (LDA score >3.0 or <-3.0, *p < *0.05). *, *p < *0.05; **p < 0.01; ***p < 0.001. TC, thyroid cancer; HC, healthy control; PCoA, principal coordinate analysis; OTU, operational taxonomic units; LDA, linear discriminant analysis; LEfSe, LDA effect size.

According to the PCoA analysis in the previous section, although the difference between the samples was greater than that within the group, the *R* value in the group analysis was very small. By partitioning around medoids algorithm, individuals can be robustly clustered into two enterotypes (type 1, n=64; type 2, n=44) (**Supplementary Figure 1A**), and their distribution of sample differences is shown in **Figure 2A**. Type 1 comprised 74% of TC patients, but the ratio of the two types were almost equal in HCs (**Figure 2B**). Moreover, at the phylum level, significant differences were only observed in the abundance of *Proteobacteria* (higher in type 1, *p* < 0.001, **Figure 2C**). At the genus level, *Bacteroides, Ruminococcus_2, Ruminococcaceae_UCG-013* were enriched in type 1, and *Prevotella_9* and *Ruminococcaceae_UCG-002* had greater enrichment in type 2 (*p* < 0.05, **Figures 2D, E**).

### Certain Differential Bacteria In TC Patients

Next, we performed preliminary discriminant analysis on bacterial community structure at different taxonomic levels to identify the differentially abundant taxa in the TC group. At the phylum level, the abundance of *Proteobacteria* was higher in the TC group than in HCs (*p* < 0.05, **Figures 2F, G**). Then, we calculated the ratio of *Firmicutes*/*Bacteroidetes*, and there were no differences in the ratio between the two groups (**Supplementary Figure 1B**). Meanwhile, we did not find significant correlations between the ratio and age or BMI in either group (**Supplementary Figures 1C, D**). At the genus level, LEfSe analysis demonstrated that a total of 25 changed genera could be regarded as biomarkers to distinguish TC patients from HCs (seven dominant genera in TC; 18 in HC, **Figure 2H**). In addition, the LEfSe results demonstrated that the *g_Christensenellaceae_R-7_group* (LDA score = 3.3, *p* < 0.001) and *g_[Eubacterium]_coprostanoligenes_group* (LDA score = 3.5, *p* < 0.001) levels were significantly decreased in the TC group (**Figure 2H**).

### Microbial Prediction Model Associated With TC Patients

**Figure 3:**
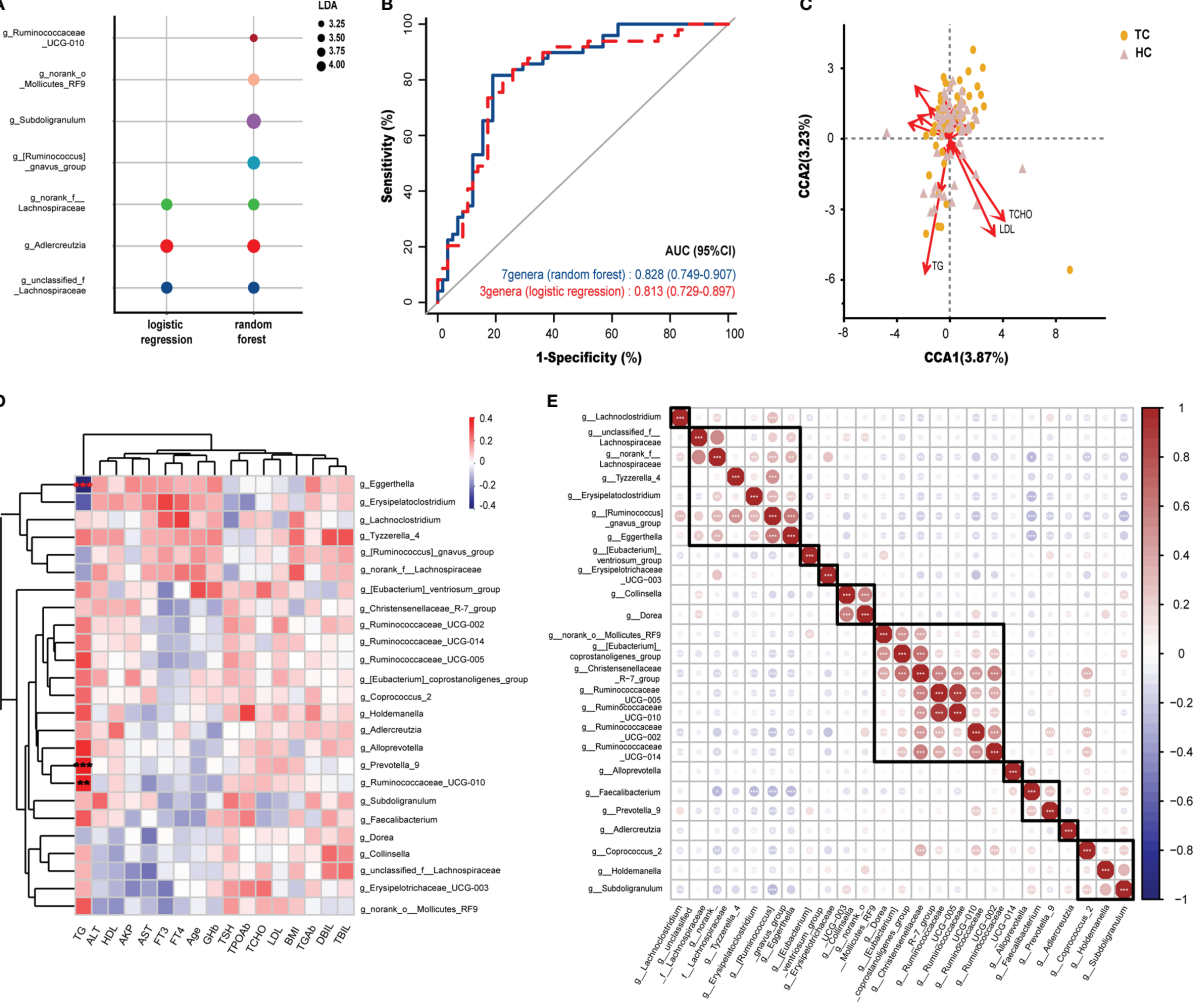
Potential diagnostic markers of gut microbiota in TC patients and Spearman correlation in environmental factors and microbiota. **(A)** The composition of genera according to the forward stepwise logistic regression and random forest model. **(B)** ROC curve analysis revealed the effect of the above two models by the AUCs. **(C)** CCA results of gut microbiota and clinical factors of patients with TC and HC group. **(D)** Spearman correlation between environmental factors and 25 genera based on LEfSe analysis results. **(E)** Spearman correlation and ward hierarchical clustering (n = 10) of 25 genera based on LEfSe analysis results. *, *p < *0.05; **p < 0.01; ***p < 0.001. TC, thyroid cancer; HC, healthy control; ROC, receiver operator characteristic; AUC, areas under the curve; CCA, Canonical correspondence analysis; LDA, linear discriminant analysis; LEfSe, LDA effect size; BMI, body mass index; fT3, free triiodothyronine; fT4, free thyroxine; TSH, thyroid-stimulating hormone; TGAb, thyroglobulin antibody; TPOAb, thyroid peroxidase antibody; AKP, alkaline phosphatase; DBil, direct bilirubin; TBil, total bilirubin; ALT, alanine aminotransferase; AST, aspartate aminotransferase; GHb, glycated hemoglobin; TCHO, total cholesterol; TG, triglycerides; HDL, high density lipoprotein; LDL, low density lipoprotein.

According to the 25 altered genera identified by LEfSe analysis, we subsequently explored novel microbial predictors for noninvasive screening and diagnosis of TC. Lasso regression validated that 23 genera could be used as combined markers. Three genera were indicated in forward stepwise logistic regression, and seven genera were indicated in the random forest model (**Supplementary Table 1**, **Supplementary Figures 2A–C**). As we preferred to include relatively few floras to set up the prediction model, we did not include the results of the lasso regression in the following research. Then, we compared the composition of genera between the other two analyses (**Figure 3A**). Based on the ROC curve, the AUCs (95% CI) of the two models were 0.828 (0.75-0.91, random forest), 0.813 (0.73-0.90, logistic regression)(**Figure 3B**). However, there were no significant differences among the pairwise comparisons of the AUCs of each model (*Z* test, *p* > 0.05; **Supplementary Table 2**).

### Correlations Between the Intestinal Flora and Clinical Characteristics

To determine the influence of clinical factors on our sample distribution, CCA was performed. After filtering with variance inflation factor analysis, the CCA results shown in **Figure 3C** demonstrated that TGs, TCHO and LDL had the strongest contribution to the sample distribution (*r^2^* > 0.176, *p* < 0.01, **Supplementary Table 3**). Furthermore, Spearman correlation analysis was performed between 25 differential genera (by LEfSe analysis) and 17 clinical characteristics. It was obvious that the relationships between serum TG levels and differential genera were completely different from other clinical indicators at the genus level (**Figure 3D**). TG was significantly positively correlated with *Prevotella_9* (r = 0.33, p < 0.001) and *Ruminococcaceae_UCG-010* (*r* = 0.31, *p* < 0.01) and significantly negatively correlated with *Eggerthella Igolzaea* (*r* = -0.40, *p* < 0.001).

All intestinal bacteria constitute a complex ecosystem, where microorganisms are influenced not only by the physiological activities of the host but also by other genera in the microecological environment. Therefore, Spearman correlation was analyzed on the 25 genera that were altered between TCs and HCs. We found 10 clusters of flora with similar expression patterns in our samples by clustering. Interestingly, *Christensenellaceae_R-7_group*, which was found in one of the clusters, had a strong correlation with *[Eubacterium]_coprostanoligenes_group* (*r* > 0.60, *p* < 0.001) and five other genera within the same cluster (**Figure 3E**), which indicated a strong coexistence among them.

### Metabolic Features in the TC and HC Groups

**Figure 4:**
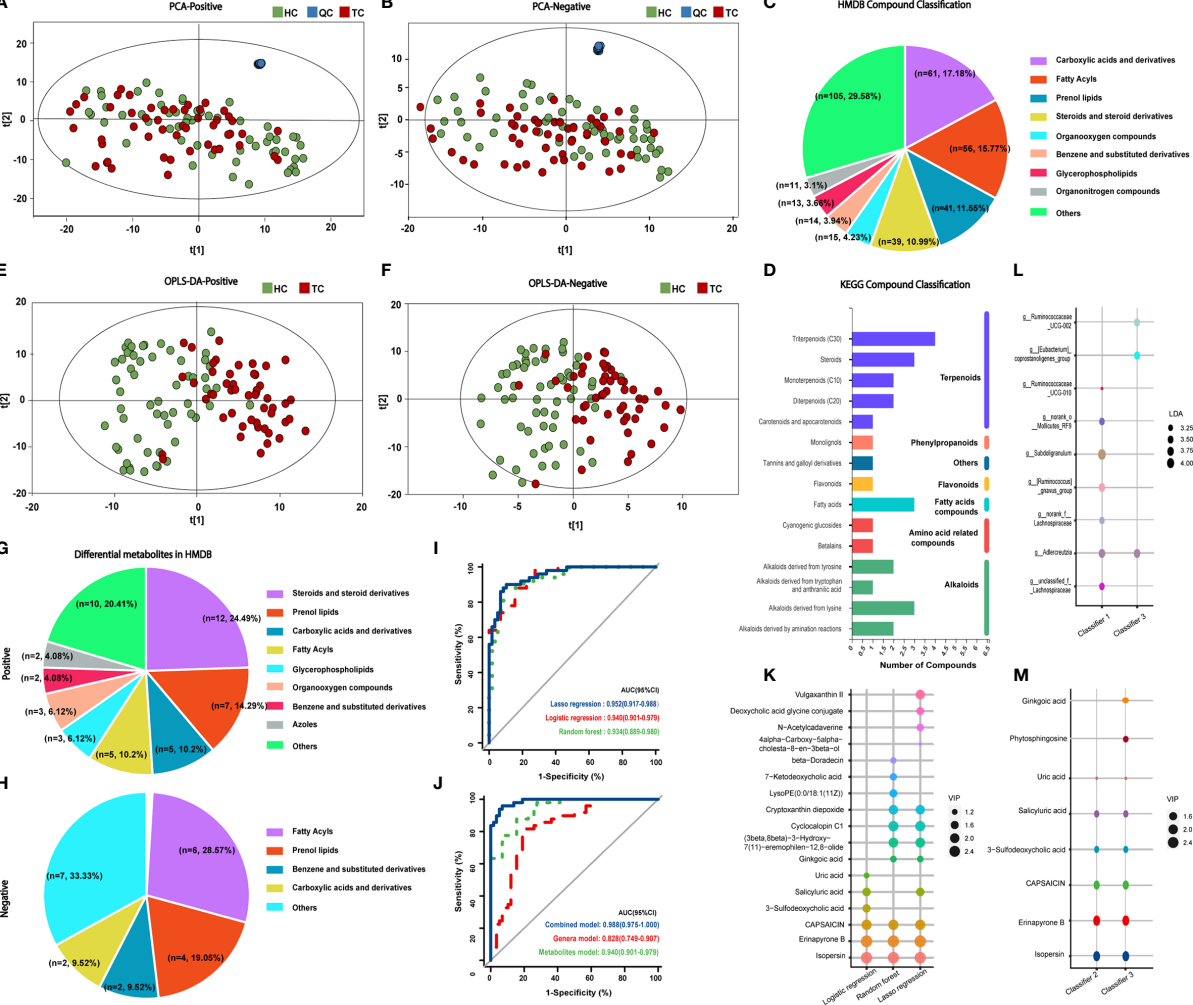
Classification of metabolites in TC patients and HCs and prognostic models of metabolites. PCA results of **(A)** positive and **(B)** negative compounds in TC and HC groups. Annotation and proportion of metabolites based on the **(C)** HMDB and **(D)** KEGG databases. OPLS-DA results of **(E)** positive and **(F)** negative compounds in TC and HC groups. Annotation and proportion of differential metabolites from OPLS-DA results in **(G)** HMDB and **(H)** KEGG databases. **(I)** ROC curve analysis revealed the effect of the three models correlated with metabolites by the AUCs. **(J)** ROC curve analysis revealed the effect of the genus model of random forest (classifier I, 7 genera), the metabolite model of logistic regression (classifier II, 6 metabolites) and combined model (classifier III, 3 genera and 8 metabolites) of logistic regression by the AUCs. **(K)** The composition of metabolites according to the forward stepwise logistic regression, lasso regression and random forest model. **(L, M)** The composition of genera and metabolites among classifier I, II and III. TC, thyroid cancer; HC, healthy control; QC, quality control; PCA, Principal Component analysis; HMDB, Human Metabolome Database; KEGG, Kyoto Encyclopedia of Genes and Genomes; OPLS-DA, orthogonal partial least squares discrimination analysis; ROC, receiver operator characteristic; AUC, areas under the curve; LDA, linear discriminant analysis; VIP, variable importance in projection.

To evaluate whether the profiles of metabolites were related to TC, we performed metabolic profiling in all fecal samples. In our study, 4385 cationic ions (363 metabolites identified) and 4453 anionic ions (161 metabolites identified) were obtained (**Supplementary Material**). As shown in **Figures 4A, B**, the differences in classification between TC patients and HCs were not obvious; there were few differences in composition between the two groups. Since evidence from this study indicated that the intestinal flora might affect the metabolism of fatty acids, we subsequently focused on fatty acid metabolites.

HMDB annotation results identified 56 fatty acyl groups, 39 sterols and their derivatives (**Figure 4C**); KEGG annotation results identified three fatty acid compounds (**Figure 4D**). OPLS-DA analysis (**Figures 4E, F**, **Supplementary Figures 3A, B**) identified a total of 49 differential positive compounds involved in human metabolism between the TC and HC groups, and 21 negative compounds were identified (variable importance in projection (VIP) > 1.0, *p* < 0.05, **Tables 2**, **3**). The dominant differential metabolites of positive and negative cations that were indicated by the HMDB database are shown in **Figures 4G, H**.

**Table 2:** Detailed information on differential cation metabolites.

Compound	Measuredm/z (Da)	Theoretical m/z (Da)	RT (min)	Mass error (ppm)	HMDB ID	VIP value	Fold change	*P* value^†^
Flazine	309.08701	309.08742	3.878	-1.34278	HMDB0033459	2.800	0.74	<0.001
Erinapyrone B	184.09599	184.09576	2.831	1.26539	HMDB0041427	2.718	1.26	<0.001
Isopersin	403.28201	403.28159	8.615	1.05430	HMDB0032735	2.647	1.71	<0.001
4-Butyloxazole	126.09100	126.09107	2.913	-0.53850	HMDB0038288	2.639	1.48	0.002
N-Acetyl-L-phenylalanine	190.08600	190.08626	3.302	-1.34945	HMDB0000512	2.636	1.28	<0.001
4,5-Dimethyloxazole	139.08600	139.08623	2.514	-1.66307	HMDB0032970	2.426	1.36	0.004
Capsaicin	306.20599	306.20605	5.893	-0.17490	HMDB0002227	2.219	0.54	<0.001
(3beta,8beta)-3-Hydroxy-7 (11)-eremophilen-12,8-olide	292.19101	292.19101	5.717	0.00983	HMDB0040754	2.077	0.79	<0.001
N-desmethylclarithromycin	698.44598	698.44680	6.465	-1.17235	HMDB0061020	2.067	0.74	0.001
Berberine	336.12299	336.12268	3.129	0.89722	HMDB0003409	2.036	1.26	0.004
Genipic acid	226.10899	226.10864	2.491	1.54579	HMDB0036072	2.001	0.73	<0.001
Aminocaproic acid	173.12801	173.12816	1.380	-0.91612	HMDB0001901	1.976	1.33	0.002
PI (20:2 (11Z,14Z)/18:2 (9Z,12Z))	887.56500	887.56425	11.787	0.84837	HMDB0009877	1.974	1.24	0.024
Valyl-Hydroxyproline	213.12300	213.12334	0.737	-1.58757	HMDB0029128	1.970	0.65	0.003
Cryptoxanthin diepoxide	585.42798	585.43020	8.544	-3.79946	HMDB0029896	1.948	0.81	<0.001
27-hydroxycholesterol	425.33701	425.33858	6.808	-3.71261	HMDB0002103	1.899	0.82	0.001
Polypropylene glycol (m w 1,200-3,000)	117.09100	117.09095	4.517	0.46124	HMDB0032478	1.892	0.64	0.001
1-[(5-Amino-5-carboxypentyl) amino]-1-deoxyfructose	273.14401	273.14417	0.648	-0.57041	HMDB0034879	1.825	0.79	0.027
Cholesterol	409.34601	409.34437	8.278	3.99226	HMDB0000067	1.825	0.75	0.007
3,4-octadienoylglycine	230.14000	230.13977	2.248	1.01843	HMDB0094791	1.821	0.82	0.003
Lotaustralin	303.15500	303.15494	0.638	0.18435	HMDB0033865	1.791	0.92	0.015
3-Sulfodeoxycholic acid	473.25699	473.25653	5.831	0.95933	HMDB0002504	1.651	1.28	0.017
6- (alpha-D-Glucosaminyl)-1D-myo-inositol	342.13901	342.13942	0.618	-1.19749	HMDB0011668	1.633	0.86	0.015
Lucyoside Q	635.41199	635.41439	6.553	-3.78093	HMDB0029621	1.629	0.90	0.005
LysoPE (0:0/18:1 (11Z))	512.33301	512.33429	9.154	-2.50468	HMDB0011475	1.582	1.37	0.022
Deoxycholic acid glycine conjugate	899.63599	899.63519	6.144	0.88151	HMDB0000631	1.565	0.83	0.001
Fasciculol C	509.38400	509.38397	8.123	0.06533	HMDB0035853	1.563	0.92	0.015
Solasodine	414.33600	414.33625	4.943	-0.61162	HMDB0035282	1.560	0.71	0.010
Ikshusterol	431.38800	431.38841	9.934	-0.93950	HMDB0030022	1.550	0.88	0.002
Linoelaidic Acid	561.48700	561.48873	8.937	-3.09053	HMDB0006270	1.543	1.08	0.024
7-Dehydrocholesterol	385.34601	385.34643	10.407	-1.08787	HMDB0000032	1.520	0.91	0.003
DG (16:0/18:0/0:0)	638.57202	638.57225	8.948	-0.36012	HMDB0007100	1.499	1.07	0.037
N-Acetylcadaverine	145.13300	145.13302	1.008	-0.15069	HMDB0002284	1.466	1.22	0.001
2-Hydroxybenzaldehyde	164.07001	164.07061	2.981	-3.64974	HMDB0034170	1.464	1.13	0.003
Cer (d18:0/16:0)	562.51801	562.51688	8.948	1.99729	HMDB0011760	1.462	1.06	0.006
(3beta,15alpha,22S,24E)-3,15,22-Trihydroxylanosta-7,9 (11),24-trien-26-oic acid	487.34100	487.34218	6.597	-2.42396	HMDB0035295	1.462	0.94	0.003
Styrene	105.07000	105.06973	2.270	2.52180	HMDB0034240	1.459	1.30	0.021
Gamma-Glutamyltyrosine	275.10300	275.10239	2.774	2.18931	HMDB0011741	1.454	0.81	0.039
Phytosphingosine	381.31100	381.30984	5.717	3.05661	HMDB0004610	1.449	0.89	0.029
Mangiferic acid	245.22600	245.22606	7.484	-0.24932	HMDB0029800	1.421	1.13	0.002
Beta-Doradecin	625.35797	625.35987	3.699	-3.03585	HMDB0039143	1.280	0.86	0.032
1-Hexanol	144.13800	144.13817	4.866	-1.15216	HMDB0012971	1.267	0.84	0.015
PE (16:0/P-18:1(11Z))	666.52698	666.52534	9.154	2.45727	HMDB0008951	1.199	0.94	0.043
16-Ketoestradiol	287.16400	287.16461	4.506	-2.12684	HMDB0000406	1.194	0.80	0.027
3-Oxocholic acid	407.27899	407.27907	4.889	-0.19822	HMDB0000502	1.173	1.08	0.024
Collettiside I	621.32300	621.32170	3.655	2.08461	HMDB0029310	1.075	1.04	0.007
4alpha-Carboxy-5alpha-cholesta-8-en-3beta-ol	453.33600	453.33617	7.418	-0.37620	HMDB0012166	1.075	0.96	<0.001
7a,12a-Dihydroxy-3-oxo-4-cholenoic acid	405.26300	405.26371	4.495	-1.75785	HMDB0000447	1.072	1.07	0.020
Maslinic Acid	473.36099	473.36295	7.527	-4.13426	HMDB0002392	1.058	0.97	0.016

^†^Comparisons between TC patients and HCs.

DG, diacylglycerol; HC, healthy control; HMDB, Human Metabolome Database; m/z, mass-to-charge ratio; PI, phosphatidylinositol; RT, retention time; TC, thyroid cancer; VIP, variable importance in projection. (VIP values >1, p <0.05).

**Table 3:** Detailed information on differential anion metabolites.

Compound	Measuredm/z (Da)	Theoreticalm/z (Da)	RT (min)	Mass error (ppm)	HMDB ID	VIP value	Foldchange	*P* value^†^
3-O-cis-Coumaroylmaslinic acid	663.38898	663.39000	7.872	-1.54804	HMDB0034539	2.871	0.81	0.005
1,11-Undecanedicarboxylic acid	487.33200	487.33334	5.721	-2.75012	HMDB0002327	2.743	0.80	<0.001
Cyclocalopin C1	295.11899	295.11894	3.496	0.14898	HMDB0039812	2.207	0.59	0.001
1-Isopropyl citrate	513.14600	513.14607	0.748	-0.13513	HMDB0032438	2.166	0.68	0.002
Vulgaxanthin II	385.09201	385.09081	3.051	3.12724	HMDB0029841	1.977	1.29	<0.001
xi-10-Hydroxyoctadecanoic acid	345.26300	345.26451	8.830	-4.36486	HMDB0037396	1.954	0.84	0.013
Actinidic acid	531.33301	531.33303	7.872	-0.05049	HMDB0037963	1.912	0.86	0.030
(1’R)-Nepetalic acid	229.10800	229.10863	3.911	-2.73023	HMDB0036117	1.848	1.35	0.036
Tetradecanedioic acid	257.17499	257.17556	5.983	-2.20854	HMDB0000872	1.771	0.88	0.043
Methyl 7-epi-12- hydroxyjasmonate glucoside	423.16000	423.16028	0.690	-0.65586	HMDB0031763	1.752	0.84	0.025
Salicyluric acid	194.04601	194.04590	2.453	0.53013	HMDB0000840	1.710	0.79	0.006
Lucidenic acid H	497.24799	497.24869	3.006	-1.41305	HMDB0035908	1.649	0.88	0.038
Ricinoleic acid	297.24301	297.24391	7.500	-3.02021	HMDB0034297	1.510	1.06	0.006
7-Ketodeoxycholic acid	451.26901	451.27065	4.598	-3.62833	HMDB0000391	1.419	1.06	0.028
(1xi,3xi)-1,2,3,4-Tetrahydro-1-methyl-beta-carboline-3-carboxylic acid	229.09900	229.09797	2.579	4.49806	HMDB0037942	1.356	0.93	0.003
Ginkgoic acid	691.49298	691.49409	9.500	-1.60630	HMDB0033897	1.318	0.94	<0.001
Uric acid	167.02200	167.02148	0.979	3.12724	HMDB0000289	1.200	0.89	0.021
Docosapentaenoic acid (22n-6)	375.25400	375.25376	8.830	0.64032	HMDB0001976	1.194	1.06	0.012
Enterodiol	301.14401	301.14493	3.279	-3.03912	HMDB0005056	1.143	0.84	0.009
PE (15:0/22:1(13Z))	804.57300	804.57624	11.322	-4.02805	HMDB0008908	1.117	1.10	0.046
DG (14:0/22:5(7Z,10Z,13Z,16Z, 19Z)/0:0)	659.48499	659.48695	10.600	-2.97927	HMDB0007033	1.084	0.95	0.009

^†^Comparisons between TC patients and HCs.

DG, diacylglycerol; HC, healthy control; HMDB, Human Metabolome Database; m/z, mass-to-charge ratio; PE, phosphatidylethanolamine; RT, retention time; TC, thyroid cancer; VIP, variable importance in projection. (VIP values >1, p < 0.05).

### Predictive Model of Specific Intestinal Metabolites and the Combined Model

On the basis of the 70 differential metabolites between the two groups that were identified in the previous section, three methods were employed to construct metabolite models for TC prediction (**Supplementary Table 4** and **Supplementary Figures 3C–E**). The metabolites included in the three models and the diagnostic effectiveness of the three models are shown in **Figures 4K, I**. These results indicate that the stepwise logistic regression classifier shows great predictive ability with relatively few metabolites (classifier II).

Next, we entered all 25 genera and 70 metabolites with apparent distinction between the TC and HC groups into forward stepwise logistic regression (classifier III, combination of genus and metabolites, **Supplementary Table 5**). The distinguishing ability of the models among genera (classifier I, the composition of seven differential genera generated by random forest model analysis), metabolites (classifier II) and their combination (classifier III, **Figures 4L, M**) was compared. *Adlercreutzia* was the only same genus between classifiers I and III, and the common metabolites between classifiers II and III were isopersin, erinapyrone B, 3-sulfodeoxycholic acid, capsaicin, salicyluric acid and uric acid. Moreover, as shown in **Figure 4J** and **Supplementary Table 6**, the metabolite model (AUC = 0.940, 95% CI: 0.90-0.98) was significantly better than the microbial model (AUC = 0.828, 95% CI: 0.75-0.91, *Z* = 2.504, *p* = 0.012). Additionally, the combined model (AUC = 0.967, 95% CI: 0.93-1.00) was markedly better than the microbial model (*Z* = 3.198, *p* = 0.001). However, there was no greater distinction between the combined model and the metabolite model (*Z* = 1.029, *p* = 0.304). Subsequently, the above results were validated by an additional validation cohort (TC vs HC, n = 20 and n = 20, **Supplementary Figure 4**), indicating the accuracy of our predictive models.

### Functional Enrichment Pathway of Metabolites in TC Patients

**Figure 5:**
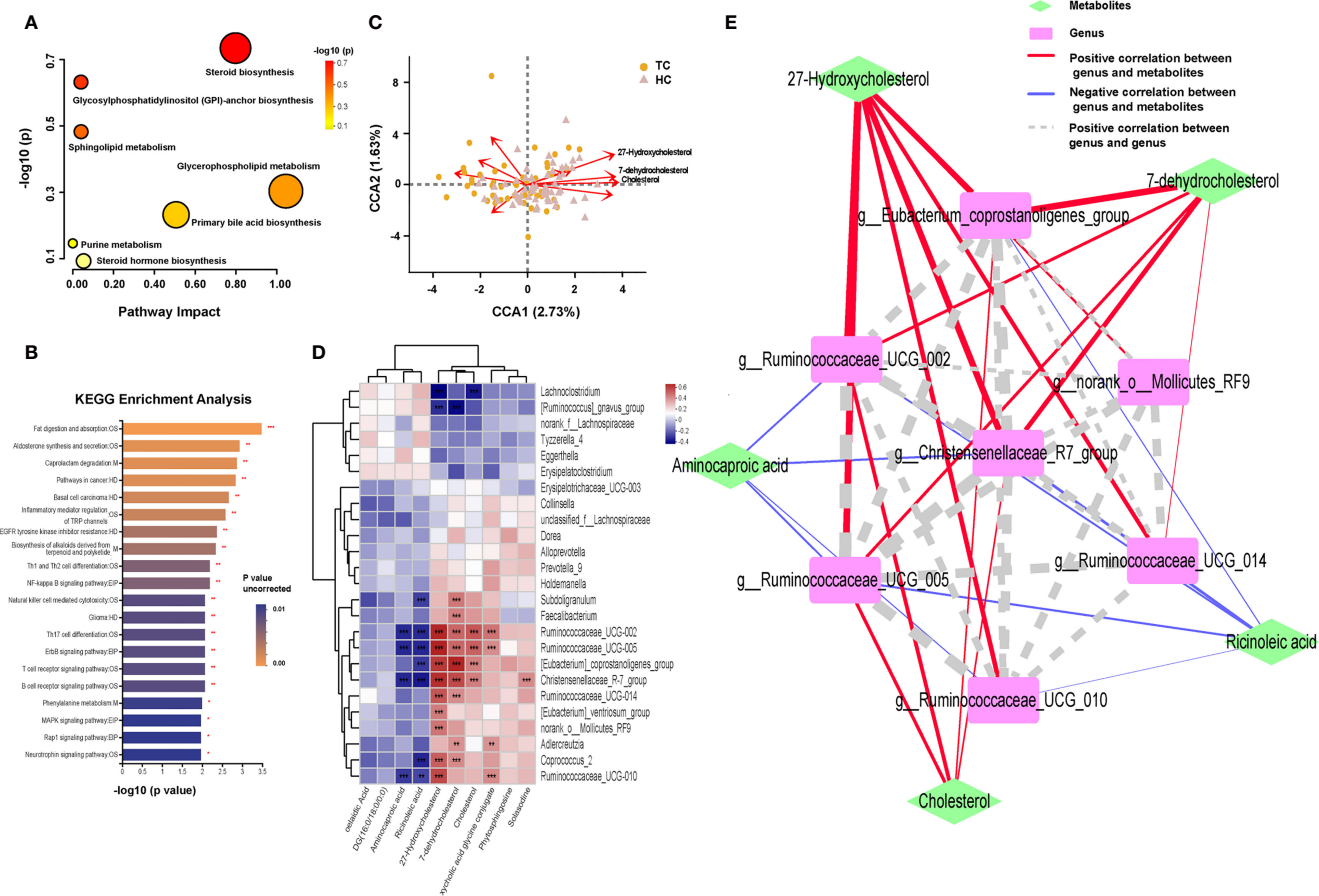
Enrichment of function pathway and correlation between metabolites and microbiota in TC patients and HCs. Metabolic pathways in **(A)** MetaboAnalyst platform (*p *> 0.05 of all) and **(B)** Major Cloud platform. **(C)** CCA results of gut microbiota and 10 differential lipid metabolites of patients with TC and HC group. **(D)** Spearman correlation between 10 differential lipid metabolites and 25 differential genera based on the LEfSe analysis results. **(E)** Network Diagram based on the Spearman correlation showed the relationship between 5 core differential lipid and 7 core differential genera. *, *p < *0.05; **p < 0.01; ***p < 0.001. TC, thyroid cancer; HC, healthy control; CCA, canonical correspondence analysis; LEfSe, linear discriminant analysis effect size; DG, diacylglycerol; OS, organismal systems; M, metabolism; HD, human disease; EIP, environmental information processing.

Next, we aimed to explore the functional influence of these metabolites and characterize which metabolic pathways they might be related to. There were seven enriched metabolic pathways in the MetaboAnalyst platform with no statistical significance (*p* > 0.05 of all, **Supplementary Table 7** and **Figure 5A**), and 66 networks in the Major Cloud platform were determined to be significantly different by the KEGG pathway enrichment analysis (*p* < 0.05, **Supplementary Table 8** and **Figure 5B**). The pathways related to steroid synthesis enriched by the metabolite cholesterol were highly significant in both platforms. For example, cholesterol and 7-dehydrocholesterol were enriched in the steroid biosynthesis [M00101] pathway in the MetaboAnalyst platform (*p* = 0.184, corrected *p* > 0.05), and cholesterol and DG (16:0/18:0/0:0) showed strong enrichment in the pathway of fat digestion and absorption by the Major Cloud platform (corrected *p* = 0.028). Notably, the abundance of metabolic compounds (cholesterol, 7-dehydrocholesterol, DG (16:0/18:0/0:0) enriched in the above pathways was decreased in the TC group. These results suggest that functional pathways, such as steroid biosynthesis and lipid digestion, might be inhibited, which would further lead to the dysfunction in the TC group.

### Correlation and Network Analysis of Metabolites and the Microbiota

Based on the above results, we subsequently analyzed the potential association between genera and metabolic compounds, especially lipid metabolites. There were a total of 10 lipid compounds among 70 differential metabolites that were identified using the KEGG pathway analysis of the Major Cloud platform (**Supplementary Figures 5A–J**). Aminocaproic acid, ricinoleic acid, linoelaidic acid, and DG (16:0/18:0/0:0) were more abundant in TC patients; however, 27-hydroxycholesterol (27HC), cholesterol, 7-dehydrocholesterol, deoxycholic acid glycine, conjugate, phytosphingosine, and solasodine were more abundant in HCs. After the filtering with variance inflation factor analysis, the CCA results (**Figure 5C**) demonstrated that more than 10 lipid compounds significantly impacted the distribution of flora in the samples, and 27HC had the most robust influence (*r^2^* = 0.3838, *p* = 0.001), followed by cholesterol (*r^2^* = 0.2637, *p* = 0.001) and 7-dehydrocholesterol (*r^2^* = 0.2497, *p* = 0.001, **Supplementary Table 9**). Significant relationships between the TC and HC groups were uncovered by Spearman correlation analysis of 28 differential bacteria and 10 differential lipid compounds (**Figure 5D**). 27HC positively correlated with *g_Christensenellaceae_R-7_group* (*r* = 0.529, *p* < 0.001), *g_*Eubacterium_coprostanoligenes_group (*r* = 0.504, *p* < 0.001), *g_Ruminococcaceae_UCG-002* (*r* = 0.544, *p* < 0.001), and *g_Ruminococcaceae_UCG-005* (*r* = 0.557, *p* < 0.001). Then, based on the above correlations, the intestinal fecal genera and lipid compounds were further analyzed by the Spearman correlation network (**Figure 5E**). The core metabolites in both groups were 27HC and cholesterol, and the key bacteria were *g_Christensenellaceae_R-7_group*, *g_Eubacterium_coprostanoligenes_group*, *g_Rumenococcus UCG-002*, *g_Rumenococcus UCG-005*, and *g_Rumenococcus UCG-014*.

## Discussion

Disruptions of the normal intestinal microbiota have been validated in the pathogenesis of various chronic diseases, particularly obesity (24, 25). In our study, using multiomics analysis, the abundance and composition of the gut microbiota and metabolites in the TC group were noticeably altered compared with those in the controls, as demonstrated by dissimilarities in the enterotypes, predictive models and functional pathways. To further understand the network of microbial metabolites, two core genera, *g_Christensenellaceae_R-7_group* and *g_Eubacterium_coprostanoligenes_group*, were investigated. These genera were significantly correlated with disorders of lipid metabolism (27HC, cholesterol, 7-dehydrocholesterol) in TC patients.

In the diversity analysis, we found that the richness of the gut microbiota in TC patients was significantly lower than that in HCs. This outcome is consistent with a previous study in our center but is in contrast with other studies (8–10). Our subjects (50 TC, 58 HC) were from eastern and southeastern China, where the typical diet is more complex and includes vegetables, rice, beans, seafood and meat (26, 27). A study in 2015 showed that there are substantial differences in the intestinal flora among different regions and ethnic groups in China (28).

The results of the enterotype analysis indicated that *g_Bacteroides* was more abundant in type 1 in the TC group, and *g_Prevotella_9* was evenly distributed in both type 1 and type 2 in the HC group. Based on enterotypes mentioned in 2011, the abundance of *g_Bacteroides* is associated with a carnivorous diet, and the abundance of *g_Prevotella_9* is associated with a vegetarian diet. These findings are consistent with previous studies that reported that a high-fat diet was a risk factor for thyroid cancer (4, 29–31). Unfortunately, our study did not include the dietary conditions of the study subjects, and this speculation should be verified by further research. In addition, in the overview of the microbial taxonomy data, *g_Prevotella_9* was uniquely distributed in the population. Its enrichment rate was high in the HC group and relatively low in the TC group, which can be explained, in part, by the differences among people with different enterotypes.

Compared with the microbial prediction model, the metabolism model demonstrated higher test efficiency, which was consistent with the fact that the intestinal metabolites were more closely related to thyroid cancer in this microenvironment (32). However, since research on the influence of gut microbiota and metabolism in thyroid cancer is rare, further studies are required to establish a complete microenvironment diagnosis model (11).

In our metabolic pathway analysis, lipid metabolism-related pathways, which were significantly enriched in differential compounds, were indicated to be dysregulated in the TC group. Cholesterol and 27HC were significantly reduced in the TC group, while ricinoleic acid and linoelaidic acid were significantly increased in the TC group. Cholesterol is an essential part of eukaryotic cell membranes and a precursor of bile acids and steroid hormones (33). Disorders of cholesterol metabolism involve many diseases, such as arteriosclerosis, cardiovascular diseases and cancers (33). Previous studies have shown that gut microbes are closely related to cholesterol metabolism (34). Linoelaidic acid is an omega-6 unsaturated fatty acid that is rich in vegetable oil. Some studies have indicated that the diet and consumption habits in China are gradually shifting to a Western model, which leads to an increase in the proportion of omega-6 and omega-3 unsaturated fatty acids and results in obesity and inflammation (35). However, it has also been suggested that the intestinal flora can metabolize linoelaidic acid into conjugated linoelaidic acid, which has anticancer and hypolipidemic effects (36). Therefore, in situations when cholesterol and linoelaidic acid are not consumed by intestinal microbes, such as the reduced microbial diversity of the TC group observed in our study, then excessive intake of linoelaidic acid might result in disadvantages that outweigh the advantages.

The following may explain the decrease in cholesterol levels in fecal samples of the TC group. From the LEfSe analysis, we observed that *g_Christensenellaceae_R-7_group* and *g_Eubacterium_coprostanoligenes_group*, highly related to cholesterol metabolism, were significantly reduced in the TC group. Goodrich et al. found that *f_Christensenellaceae* was enriched in individuals with a normal BMI (18.5-24.9) compared to obese individuals (BMI  ≥  30) (37). Furthermore, consistent with its connection with leanness, Alemán et al. showed that *f_Christensenellaceae* was increased after diet-induced weight loss (38). It is well known that metabolic disorders are often correlated with dietary patterns (39). Additionally, *f_Christensenellaceae* has been indicated to be responsive to diet; studies have associated *f_Christensenellaceae* with the healthy dietary habits of decreased refined sugar and improved consumption of fruits and vegetables (40–42). Interestingly, our results also showed that there was a significant coexistence relationship between the *g_Christensenellaceae_R-7_group* and the *g_Eubacterium_coprostanoligenes_group*. This relationship could inhibit the reabsorption of cholesterol in the intestines by converting cholesterol to fecal sterols, so the decreased abundance may increase the intestinal reabsorption of cholesterol (34). Recent studies have reported that *g_Eubacterium_coprostanoligenes_group* can mediate the effect of a high-fat diet on host blood lipids by regulating sphingosine (43). A decrease in intestinal bacteria may affect the excretion of intestinal cholesterol in the feces and result in a decrease in the level of cholesterol measured in the feces. In fact, animal experiments that use antibiotics to destroy the intestinal flora of mice have shown that the reabsorption of cholesterol was increased in these mice (34). Therefore, the decrease in cholesterol levels in the stool of patients with thyroid cancer may be related to an increase in cholesterol reabsorption caused by the decrease in overall gut microbial diversity.

We hypothesize that long-term unhealthy diets disrupt the balance of lipid metabolism in the gut microbes in TC patients and further increase the risk factors and development of thyroid tumors. Consequently, we constructed a network relationship between floras and lipid metabolites, where 27HC demonstrated the strongest relationship with the gut microbiota in the intestinal microecology of TC patients and HCs. 27HC is an endogenous oxidized cholesterol synthesized by the liver and discovered as a selective modulator of the estrogen receptors (ERs) (44). The ERs are a key regulator of myriad physiological functions, playing a recognized or suggestive role in cancer progression in various cancer types, such as the breast, colorectal cancer and thyorid cancer (45). A previous study showed that cholesterol and 27HC could increase the aggressiveness of thyroid cancer (46). Besides, thyroid tumor samples from progressive disease were identified to have lower Oxysterol 7α-hydroxylase expression (27HC degrading enzyme) and higher concentration of 27HC (46). Many studies have also verified the association between increased 27HC and high risks of breast cancer and colorectal cancer (47). Notably, the abundance of *g_Christensenellaceae_R-7_group* in colorectal cancer is also significantly decreased, and we suspect that its deficiency promotes tumor formation (48). Thus, 27HC probably promotes the proliferation of thyroid cancer driven by the estrogen receptor and more research is needed to clarify the relationship between the *g_Christensenellaceae_R-7_group*, 27HC and thyroid tumorigenesis.

However, there are still several limitations in this study that need to be improved in the future. First, our research is a single-center, cross-sectional study, which cannot provide relevant findings on the changes in the microbiota within an individual during the development of thyroid cancer. Multicenter and prospective studies should be performed to unearth the characteristics of the intestinal microecology related to alterations in the development of the disease. Second, the accuracy and relevance of 16S rRNA gene sequencing and untargeted metabonomic analyses are restricted, and whole genome shotgun sequencing and targeted metabonomic analyses are needed to reveal the precise correlation network between floras and metabolites in thyroid cancer. Finally, our results are based on bioinformatics analyses and lack evidence to support the secondary verification results. We believe that they can be clarified by fecal microbiota transplant experiments in a thyroid cancer mouse model, and the potential coexistence of microbes can be verified by experiments in large samples worldwide.

## Conclusion

In conclusion, thyroid cancer remarkably influences the fecal microbiota and its metabolites. The *g_Christensenellaceae_R-7_group* and *g_Eubacterium_coprostanoligenes_group* were indicated as the two core genera in the microenvironment of individuals with thyroid cancer. The *g_Christensenellaceae_R-7_group* was a hub genus in the decreased genera cluster of thyroid cancer and plays an important role in maintaining the homeostasis of lipid metabolism through 27HC. In the future, further confirmation of the importance of the above core genera and metabolites in the progression of thyroid cancer is required.

## Data Availability Statement

The original contributions presented in the study are publicly available. This data can be found here: NCBI, PRJNA796956.

## Ethics Statement

The studies involving human participants were reviewed and approved by Shanghai Tenth People’s Hospital Ethics Committee (No. SHSY-IEC-KY-4.0/16-18/01). The patients/participants provided their written informed consent to participate in this study.

## Author Contributions

All authors participated in the design of the study and/or patient enrolment, and meet criteria for authorship. DL and ZL contributed to the study design, study conduct and supervision, scientific overview, data analysis, and editing of the manuscript. SF, QL and LC were directly involved in the clinic diagnosis of patients. TQ, RW, and CD collected and analyzed clinical data of subjects. GL, XY, and WJ analyzed the microbial and LM-MS data and drafted the original manuscript. The manuscript was substantively revised by DL, ZL, and XY. GL, XY and WJ contributed equally to this work and shared the co-first authorship All authors have reviewed the manuscript and approved the final version for submission

## Funding

This work was supported by the [National Natural Science Foundation of China] under Grant [number 82071964], [Shanghai Shenkang Three-year Action Project] under Grant [number SHDC2020CR2054B], [Shanghai Leading Talent Program sponsored by Shanghai Human Resources and Social Security Bureau] under Grant [number 03.05.19005], [Key discipline construction project of the three-year action plan of Shanghai public health system] under Grant [number GWV-10.1-XK9], and [Shanghai Natural Science Foundation] under Grant [number 21ZR1449600].

## Conflict of Interest

The authors declare that the research was conducted in the absence of any commercial or financial relationships that could be construed as a potential conflict of interest.

## Publisher’s Note

All claims expressed in this article are solely those of the authors and do not necessarily represent those of their affiliated organizations, or those of the publisher, the editors and the reviewers. Any product that may be evaluated in this article, or claim that may be made by its manufacturer, is not guaranteed or endorsed by the publisher.

## Supplementary Material

Supplementary Fig. 1 **(A)** When the number of clusters was 2, The CH index was the highest and the clustering effect was the best; **(B)** F/B radio in TC patients and HCs. The correlation between F/B radio and **(C)** age, **(D)** BMI. CH, Calinski-Harabasz; TC, thyroid cancer; HC, healthy control; F, Firmicutes; B, Bacteroidetes.

Supplementary Fig. 2 **(A)** The importance of the 25 differential genera was obtained by random forest analysis and expressed by Mean Decrease Accuracy. The top 7 genera were marked in red. **(B)** By 5 repetitions of ten-fold cross-validation, the error rate of cross-validation no longer decreased when the top 7 genera were selected as the model. **(C)** The dashed line on the left indicated "λ.min", which was the best λ value; the dashed line on the right indicates "λ.1se", which referred to the λ value of the simplest model obtained within a standard deviation of "λ.min".

Supplementary Fig. 3 OPLS-DA validate model and the result of random forest and lasso regression analysis in metabolites. **(A)** Validation of permutation test model in positive compounds. (n = 200). The slope of the regression line of R2 and Q2 was greater than 0, the intercept of R2 and Q2 was 0.521 and -0.360. **(B)** Validation of permutation test model in negative compounds. (n = 200). The slope of the regression line of R2 and Q2 was greater than 0, the intercept of R2 and Q2 was 0.437 and -0.353. **(C)** The importance of the 70 differential metabolites was obtained by random forest analysis and expressed by Mean Decrease Accuracy. The top 10 metabolites were marked in red. **(D)** By 5 repetitions of ten-fold cross-validation, the error rate of cross-validation no longer decreased when the top 10 metabolites were selected as the model. **(E)** The dashed line on the left indicated "λ.min", which was the best λ value; the dashed line on the right indicates "λ.1se", which referred to the λ value of the simplest model obtained within a standard deviation of "λ.min".

## Abbreviations

ALT, alanine transaminase; AST, aspartate transaminase; AUC, areas under the curve; BMI, body mass index; CCA, canonical correspondence analysis; ER, estrogen receptors; fT3, free triiodothyronine; fT4, free thyroxine; GM, gut microbiota; HC, healthy control; HDL, high-density lipoprotein cholesterol; HMDB, Human Metabolome Database; KEGG, Kyoto Encyclopedia of Genes and Genomes; LC–MS/MS, nontargeted liquid chromatography-mass spectrometry; LDA, linear discriminant analysis; LDL, low-density lipoprotein cholesterol; LEfSe, linear discriminant analysis effect size; m/z, mass-to-charge ratio; OPLS-DA, orthogonal partial least squares discrimination analysis; OTU, operational taxonomic units; PCA, principal component analysis; PCoA, principal coordinate analysis; QC, quality control; RSD, relative standard deviation; ROC, receiver operator characteristic; RT, retention time; TC, thyroid cancer; TCHO, total cholesterol; TG, triglycerides; TgAb, anti-thyroid autoantibodies; TPOAb, anti-thyroid peroxidase antibody; TSH, thyroid stimulating hormone; UPLC, ultra-performance liquid chromatography; VIP, variable importance in projection; 27HC, 27-hydroxycholesterol

## Glossary

**Table:**

ALT	alanine transaminase
AST	aspartate transaminase
AUC	areas under the curve
BMI	body mass index
CCA	canonical correspondence analysis
ER	estrogen receptors
fT3	free triiodothyronine
fT4	free thyroxine
GM	gut microbiota
HC	healthy control
HDL	high-density lipoprotein cholesterol
HMDB	Human Metabolome Database
KEGG	Kyoto Encyclopedia of Genes and Genomes
LC–MS/MS	nontargeted liquid chromatography-mass spectrometry
LDA	linear discriminant analysis
LDL	low-density lipoprotein cholesterol
LEfSe	linear discriminant analysis effect size
m/z	mass-to-charge ratio
OPLS-DA	orthogonal partial least squares discrimination analysis
OTU	operational taxonomic units
PCA	principal component analysis
PCoA	principal coordinate analysis
QC	quality control
RSD	relative standard deviation
ROC	receiver operator characteristic
RT	retention time
TC	thyroid cancer
TCHO	total cholesterol
TG	triglycerides
TgAb	anti-thyroid autoantibodies
TPOAb	anti-thyroid peroxidase antibody
TSH	thyroid stimulating hormone
UPLC	ultra-performance liquid chromatography
VIP	variable importance in projection
27HC	27-hydroxycholesterol
