# Supplementary figures and images for: Alterations of Gut Microbiome and Metabolite Profiles Associated With Anabatic Lipid Dysmetabolism in Thyroid Cancer
Source: Front Endocrinol (Lausanne). 2022 Jun 3;13:893164. doi: 10.3389/fendo.2022.893164 (PMC9204252; doi:10.3389/fendo.2022.893164)

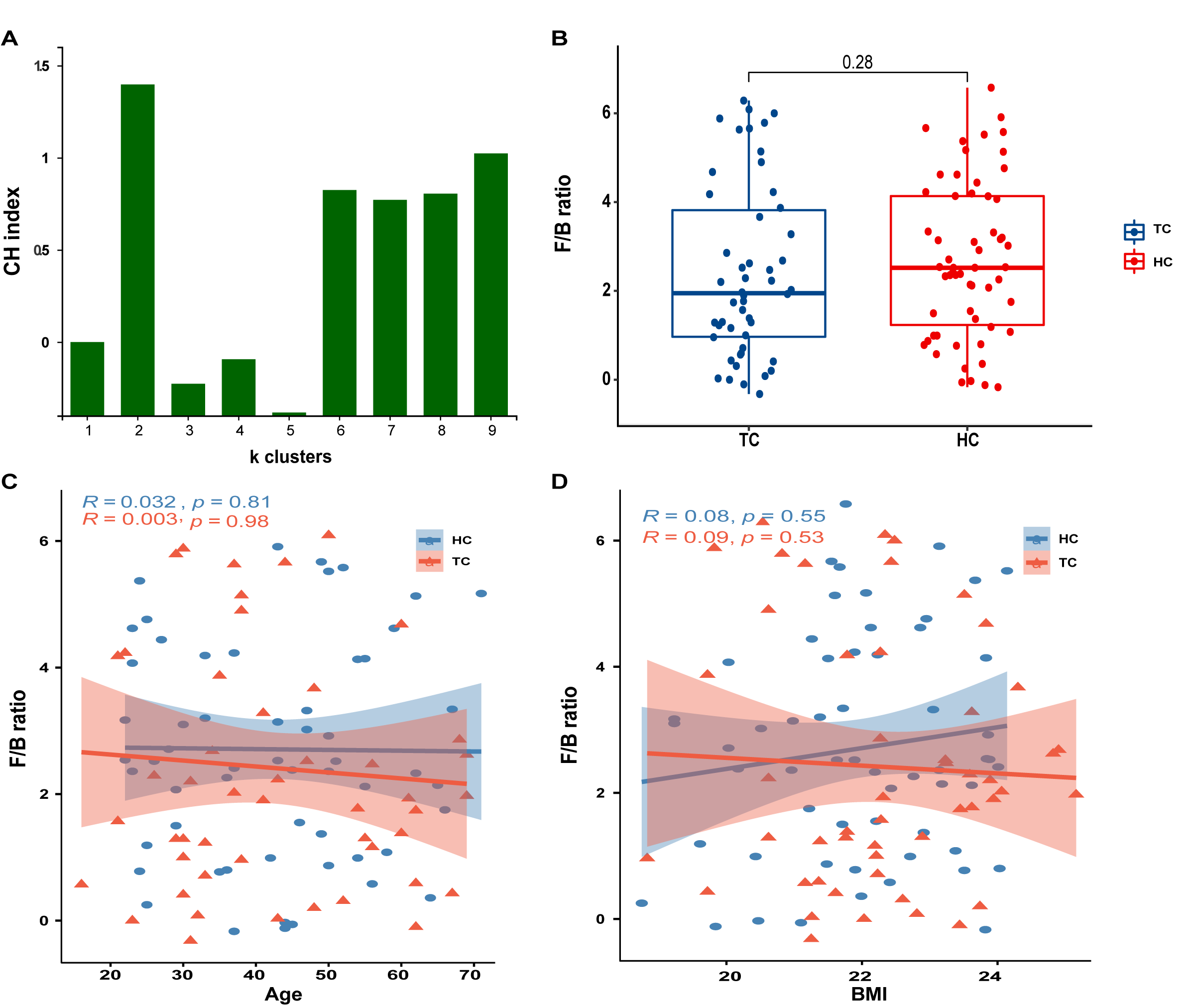

Supplement: Supplementary Fig. 1 — (A) When the number of clusters was 2, The CH index was the highest and the clustering effect was the best; (B) F/B radio in TC patients and HCs. The correlation between F/B radio and (C) age, (D) BMI. CH, Calinski-Harabasz; TC, thyroid cancer; HC, healthy control; F, Firmicutes; B, Bacteroidetes. [file Image_1.tif]

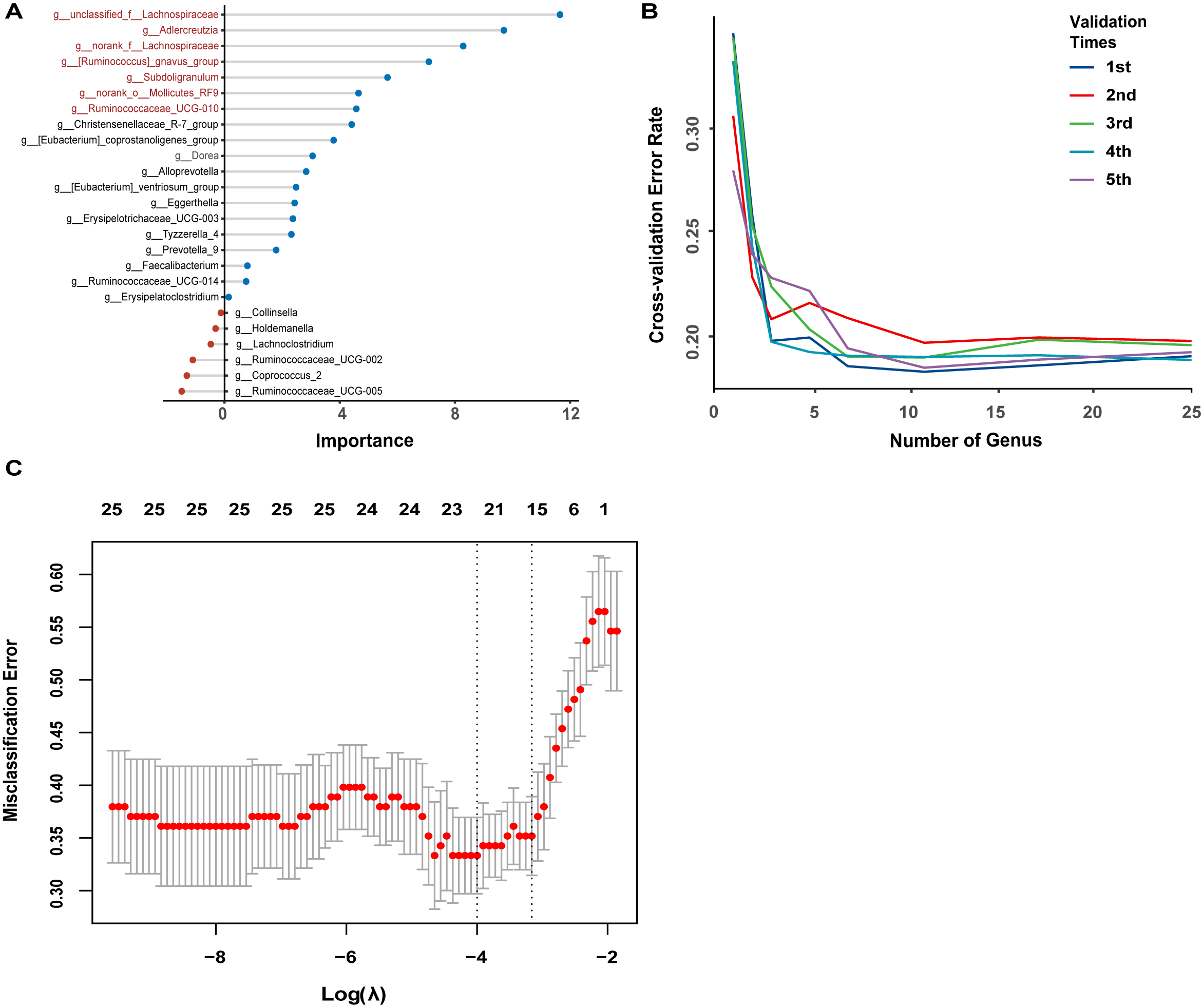

Supplement: Supplementary Fig. 2 — (A) The importance of the 25 differential genera was obtained by random forest analysis and expressed by Mean Decrease Accuracy. The top 7 genera were marked in red. (B) By 5 repetitions of ten-fold cross-validation, the error rate of cross-validation no longer decreased when the top 7 genera were selected as the model. (C) The dashed line on the left indicated "λ.min", which was the best λ value; the dashed line on the right indicates "λ.1se", which referred to the λ value of the simplest model obtained within a standard deviation of "λ.min". [file Image_2.tif]

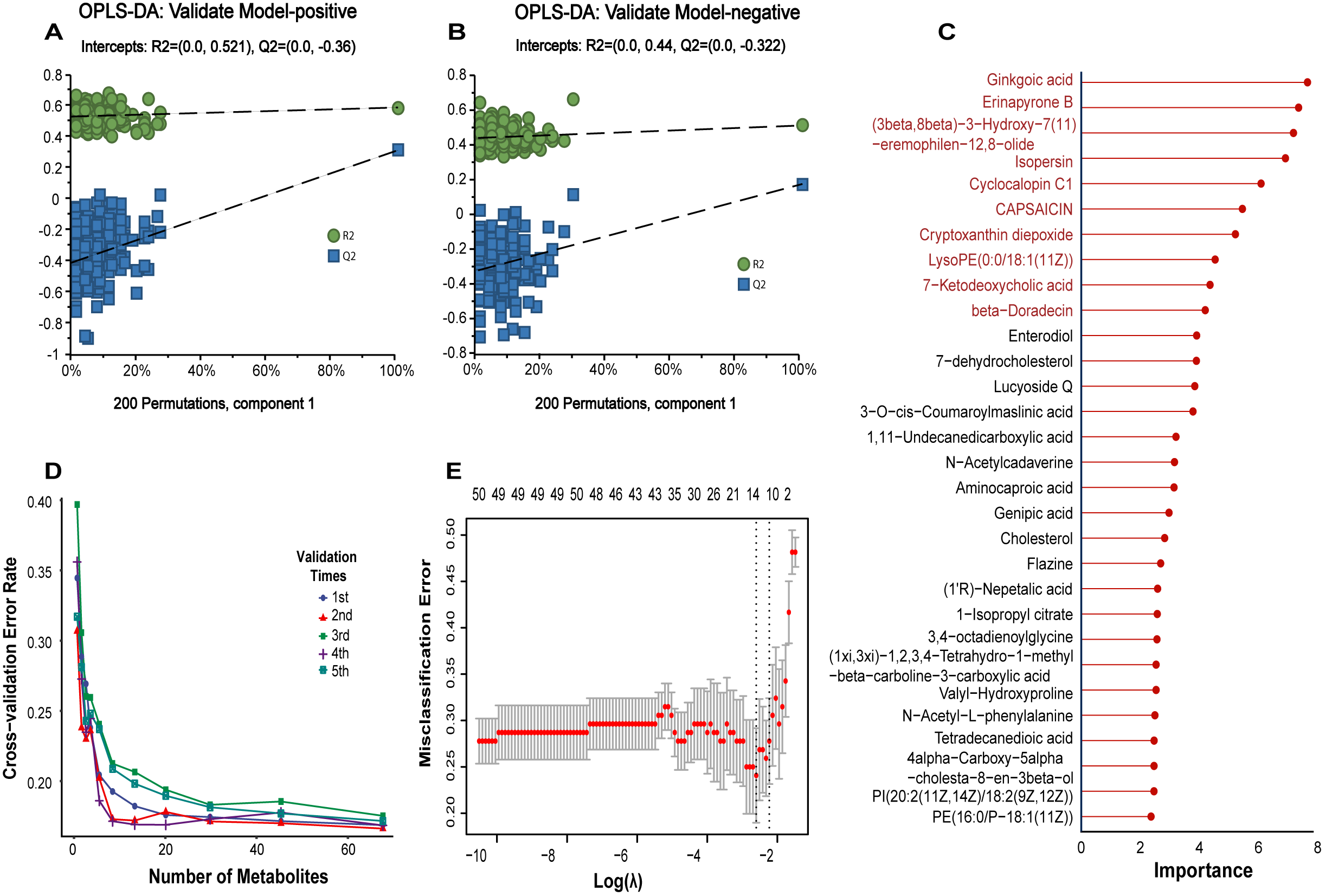

Supplement: Supplementary Fig. 3 — OPLS-DA validate model and the result of random forest and lasso regression analysis in metabolites. (A) Validation of permutation test model in positive compounds. (n = 200). The slope of the regression line of R2 and Q2 was greater than 0, the intercept of R2 and Q2 was 0.521 and -0.360. (B) Validation of permutation test model in negative compounds. (n = 200). The slope of the regression line of R2 and Q2 was greater than 0, the intercept of R2 and Q2 was 0.437 and -0.353. (C) The importance of the 70 differential metabolites was obtained by random forest analysis and expressed by Mean Decrease Accuracy. The top 10 metabolites were marked in red. (D) By 5 repetitions of ten-fold cross-validation, the error rate of cross-validation no longer decreased when the top 10 metabolites were selected as the model. (E) The dashed line on the left indicated "λ.min", which was the best λ value; the dashed line on the right indicates "λ.1se", which referred to the λ value of the simplest model obtained within a standard deviation of "λ.min". [file Image_3.tif]

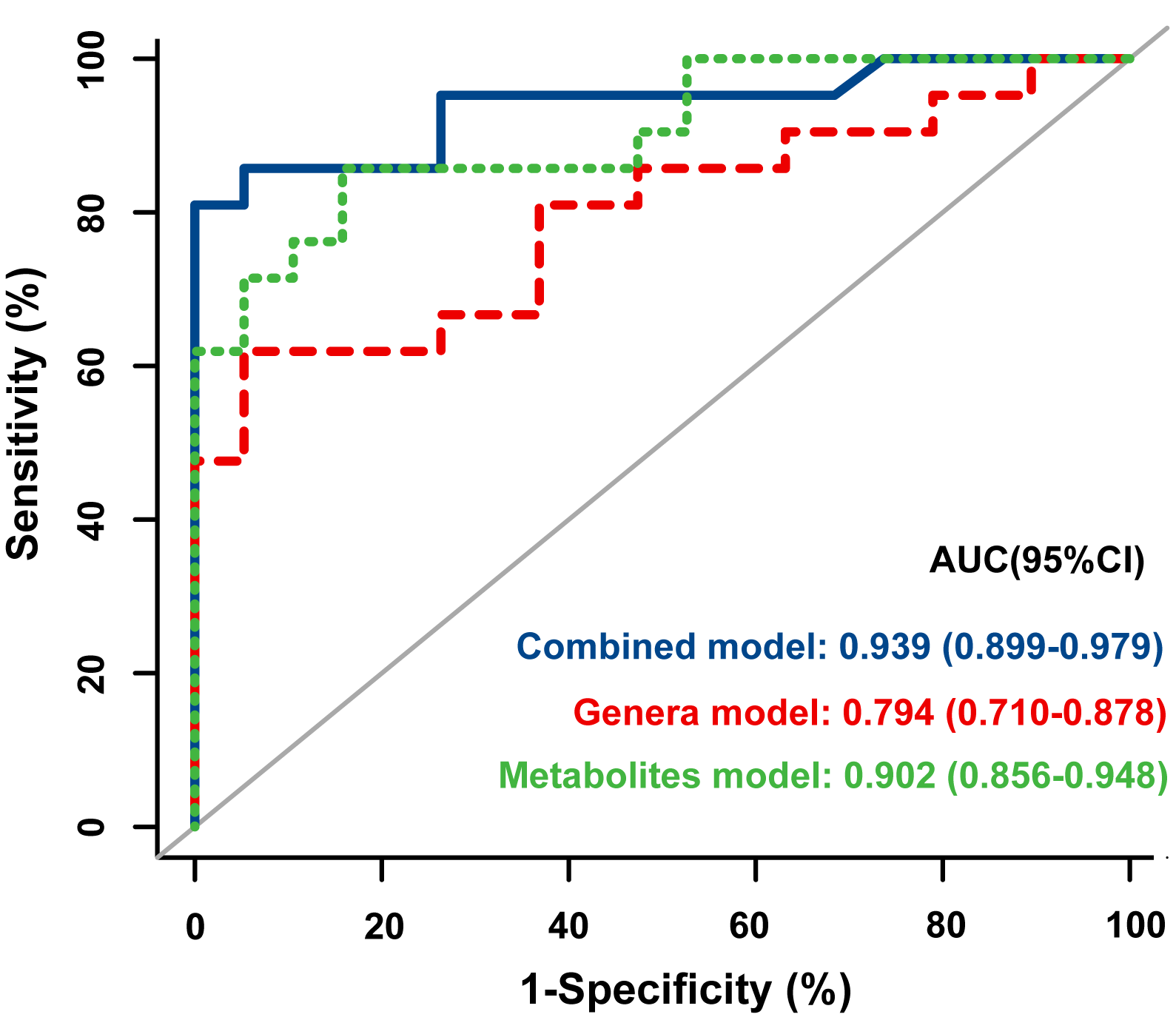

Supplement: Supplementary file 6 [file Image_4.tif]

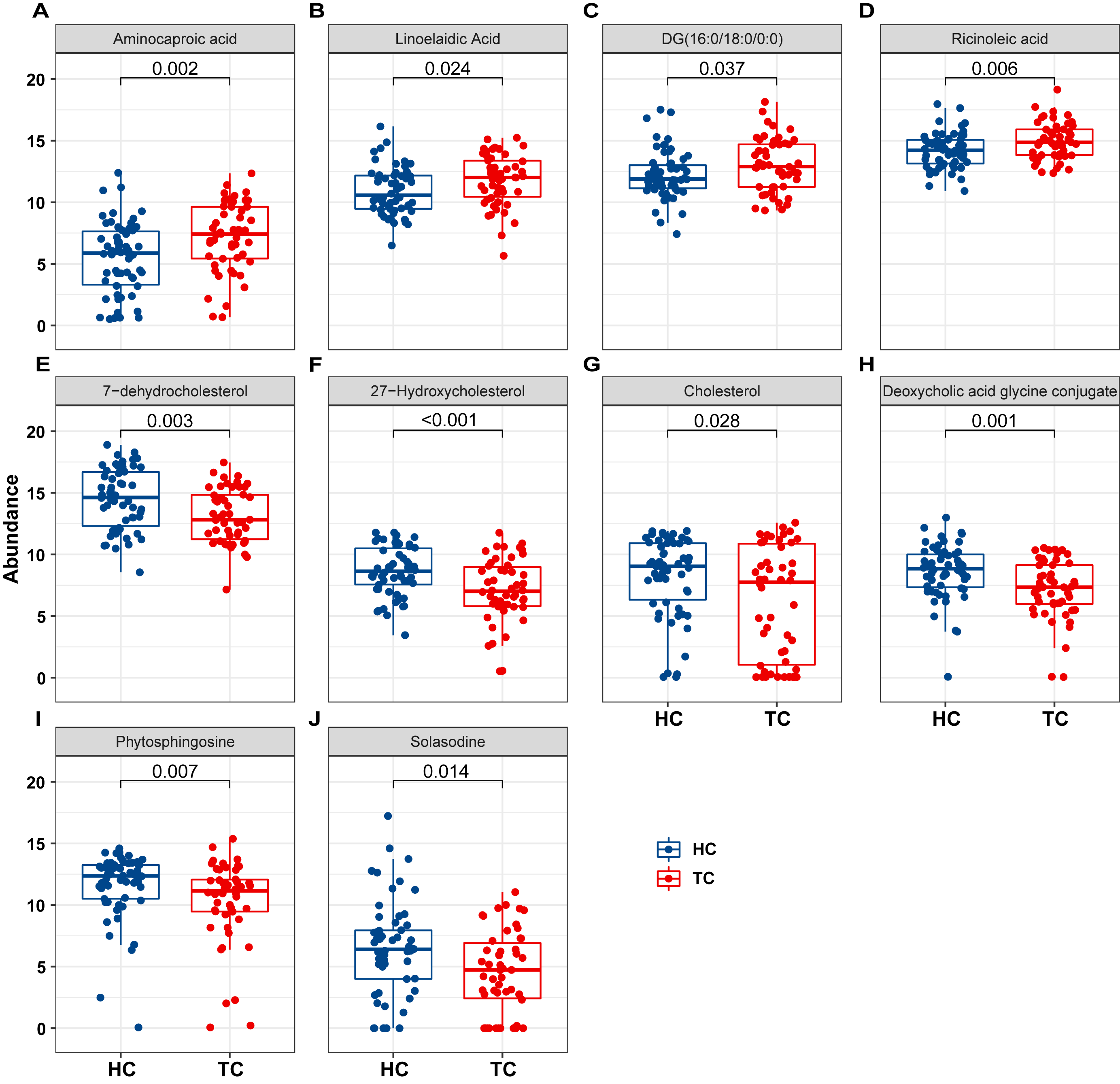

Supplement: Supplementary file 7 [file Image_5.tif]
