# Supplementary material for: Alterations of Gut Microbiome and Metabolite Profiles Associated With Anabatic Lipid Dysmetabolism in Thyroid Cancer
Source: Front Endocrinol (Lausanne). 2022 Jun 3;13:893164. doi: 10.3389/fendo.2022.893164 (PMC9204252; doi:10.3389/fendo.2022.893164)
Supplement: Supplementary file 8 [file Table_1.docx]

| **Genus** | **Sig.^*^** | **Exp(B)** | **Exp(B) 95% confidence** | |
| --- | --- | --- | --- | --- |
|  |  |  | **Lower**  **limit** | **Upper**  **limit** |
| *g_unclassified_f__Lachnospiraceae* | 0.014 | 0.998 | 0.997 | 1.000 |
| *g_Adlercreutzia* | 0.018 | 0.994 | 0.990 | 0.999 |
| *g_norank_f__Lachnospiraceae* | 0.004 | 1.005 | 1.002 | 1.009 |

**Supplemental Table 1 Genus included in the forward stepwise logistic regression**

**^*^**Comparisons between TC patients and HCs.

TC, thyroid cancer; HC, healthy control; Sig., significance.

**Supplemental Table 2 *Z* test for AUCs of pairwise models**

| **Pairwise comparison between models** | ***Z* value** | ***P* value** |
| --- | --- | --- |
| Random forest *vs* Logistic regression | 0.255 | 0.798 |
| Random forest *vs* Feng’ model | 1.264 | 0.206 |
| Logistic regression *vs* Feng’ model | 0.993 | 0.321 |

AUC, areas under the curve.

**Supplemental Table 3 CCA analysis of fecal flora and clinical factors**

| **Clinical factors** | **CCA1** | **CCA2** | ***r^2^*** | ***P* value** |
| --- | --- | --- | --- | --- |
| TCHO | 0.864 | -0.504 | 0.194 | 0.001 |
| LDL | 0.779 | -0.627 | 0.177 | 0.004 |
| TG | -0.393 | -0.920 | 0.176 | 0.002 |
| ALT | -0.862 | 0.507 | 0.075 | 0.040 |
| GHb | -0.983 | 0.183 | 0.070 | 0.059 |
| AST | -0.955 | 0.298 | 0.045 | 0.175 |
| TPOAb | -0.383 | -0.924 | 0.026 | 0.315 |
| DBIL | -0.861 | 0.508 | 0.025 | 0.358 |
| TSH | 0.986 | 0.165 | 0.016 | 0.495 |
| TBIL | -0.377 | 0.926 | 0.012 | 0.620 |
| AKP | 0.368 | 0.930 | 0.008 | 0.727 |
| TGAb | 0.731 | 0.682 | 0.005 | 0.786 |
| AGE | -0.297 | 0.955 | 0.004 | 0.857 |
| FT4 | 0.287 | 0.958 | 0.003 | 0.883 |
| HDL | -0.408 | -0.913 | 0.003 | 0.891 |
| FT3 | 0.564 | -0.826 | 0.002 | 0.938 |
| BMI | 0.469 | -0.883 | 0.001 | 0.940 |

CCA, canonical correspondence analysis; BMI, body mass index; fT3, free triiodothyronine; fT4, free thyroxine; TSH, thyroid-stimulating hormone; TGAb, thyroglobulin antibody; TPOAb, thyroid peroxidase antibody; AKP, alkaline phosphatase; DBil, direct bilirubin; TBil, total bilirubin; ALT, alanine aminotransferase; AST, aspartate aminotransferase; GHb, glycated hemoglobin; TCHO, total cholesterol; TG, triglycerides; HDL, high density lipoprotein; LDL, low density lipoprotein

**Supplemental Table 4 Metabolites included in the forward stepwise logistic regression**

| **Metabolites** | **Sig.** | **Exp(B)** | **Exp(B) 95% confidence** | |
| --- | --- | --- | --- | --- |
|  |  |  | **Lower**  **limit** | **Upper**  **limit** |
| Isopersin | 0.002 | 1.374 | 1.124 | 1.679 |
| Erinapyrone B | <0.001 | 2.099 | 1.397 | 3.152 |
| Capsaicin | <0.001 | 0.475 | 0.328 | 0.687 |
| 3-Sulfodeoxycholic acid | 0.008 | 1.369 | 1.085 | 1.728 |
| Salicyluric acid | 0.007 | 0.708 | 0.551 | 0.909 |
| Uric acid | 0.002 | 0.505 | 0.33 | 0.773 |

Sig., significance.

**Supplemental Table 5 Combination of genus and metabolites included in the forward stepwise logistic regression**

| **Genus &**  **Metabolites** | **Sig.** | **Exp(B)** | **Exp(B) 95% confidence** | |
| --- | --- | --- | --- | --- |
|  |  |  | **Lower**  **limit** | **Upper**  **limit** |
| *g__Eubacterium_coprostanoligenes_group* | 0.013 | 1.002 | 1.000 | 1.003 |
| *g__Adlercreutzia* | 0.004 | 0.976 | 0.961 | 0.992 |
| *g__Ruminococcaceae_UCG_002* | 0.016 | 0.995 | 0.991 | 0.999 |
| Isopersin | 0.004 | 2.874 | 1.388 | 5.950 |
| Erinapyrone B | 0.002 | 6.385 | 1.974 | 20.651 |
| Capsaicin | 0.002 | 0.137 | 0.039 | 0.481 |
| 3-Sulfodeoxycholic acid | 0.004 | 2.481 | 1.342 | 4.587 |
| Phytosphingosine | 0.029 | 0.480 | 0.249 | 0.926 |
| Salicyluric acid | 0.008 | 0.383 | 0.188 | 0.781 |
| Ginkgoic acid | 0.030 | 0.129 | 0.020 | 0.816 |
| Uric acid | 0.004 | 0.293 | 0.126 | 0.679 |

Sig., significance.

**Supplemental Table 6 *Z* test for AUCs of pairwise models**

| **Pairwise comparison between models** | ***Z* value** | ***P* value** |
| --- | --- | --- |
| metabolite model *vs* microbial model | 2.504 | 0.012 |
| combined model *vs* microbial model | 3.198 | 0.001 |
| combined model *vs* metabolite model | 1.029 | 0.304 |

AUC, areas under the curve.

| **Pathways** | **KEGG ID** | ***P* value^*^** | **-log10(*p*)** | **Corrected *p* value** |
| --- | --- | --- | --- | --- |
| Steroid biosynthesis | M00101 | 0.184 | 0.735 | 1 |
| GPI-anchor biosynthesis | M00065 | 0.233 | 0.632 | 1 |
| Sphingolipid metabolism | M00099/M00100 | 0.329 | 0.483 | 1 |
| Glycerophospholipid metabolism | M00091/M00092 | 0.497 | 0.303 | 1 |
| Primary bile acid biosynthesis | M00104 | 0.586 | 0.232 | 1 |
| Purine metabolism | N00888 | 0.715 | 0.146 | 1 |
| Steroid hormone biosynthesis | M00107/N00759 | 0.808 | 0.093 | 1 |

**Supplemental Table 7 Metabolic pathways enrichment in MetaboAnalyst platform**

**^*^**Comparisons between TC patients and HCs.

TC, thyroid cancer; HC, healthy control; KEGG, Kyoto Encyclopedia of Genes and Genomes; GPI, Glycosylphosphatidylinositol;

**Supplemental Table 8 Metabolic pathways enrichment in Major cloud platform**

| **Pathways** | **KEGG ID** | ***P* value^*^** | **-log10(*p*)** | **Corrected *p* value** |
| --- | --- | --- | --- | --- |
| Basal cell carcinoma | map05217 | 0.002 | 2.658 | 0.036 |
| Dopaminergic synapse | map04728 | 0.026 | 1.590 | 0.046 |
| Cholinergic synapse | map04725 | 0.026 | 1.590 | 0.046 |
| Gastric acid secretion | map04971 | 0.030 | 1.523 | 0.048 |
| Phospholipase D signaling pathway | map04072 | 0.028 | 1.556 | 0.048 |
| Amoebiasis | map05146 | 0.028 | 1.556 | 0.048 |
| Oxytocin signaling pathway | map04921 | 0.028 | 1.556 | 0.048 |
| Insulin secretion | map04911 | 0.028 | 1.556 | 0.048 |
| Adipocytokine signaling pathway | map04920 | 0.015 | 1.821 | 0.049 |
| Ras signaling pathway | map04014 | 0.015 | 1.821 | 0.049 |
| Long-term potentiation | map04720 | 0.015 | 1.821 | 0.049 |
| African trypanosomiasis | map05143 | 0.022 | 1.668 | 0.049 |
| Calcium signaling pathway | map04020 | 0.022 | 1.668 | 0.049 |
| AGE-RAGE signaling pathway in diabetic complications | map04933 | 0.022 | 1.668 | 0.049 |
| VEGF signaling pathway | map04370 | 0.013 | 1.889 | 0.049 |
| GnRH signaling pathway | map04912 | 0.013 | 1.889 | 0.049 |
| Melanogenesis | map04916 | 0.013 | 1.889 | 0.049 |
| Non-small cell lung cancer | map05223 | 0.013 | 1.889 | 0.049 |
| Phototransduction - fly | map04745 | 0.024 | 1.627 | 0.050 |
| Thyroid hormone signaling pathway | map04919 | 0.024 | 1.627 | 0.050 |
| Gap junction | map04540 | 0.024 | 1.627 | 0.050 |
| Fc epsilon RI signaling pathway | map04664 | 0.024 | 1.627 | 0.050 |
| Adrenergic signaling in cardiomyocytes | map04261 | 0.024 | 1.627 | 0.050 |
| Choline metabolism in cancer | map05231 | 0.024 | 1.627 | 0.050 |
| Circadian entrainment | map04713 | 0.024 | 1.627 | 0.050 |
| Regulation of lipolysis in adipocytes | map04923 | 0.032 | 1.493 | 0.050 |
| HIF-1 signaling pathway | map04066 | 0.032 | 1.493 | 0.050 |
| Platelet activation | map04611 | 0.032 | 1.493 | 0.050 |
| Glutamatergic synapse | map04724 | 0.017 | 1.764 | 0.050 |
| Fc gamma R-mediated phagocytosis | map04666 | 0.017 | 1.764 | 0.050 |
| Fat digestion and absorption | map04975 | 0.000 | 3.523 | 0.028 |
| Pathways in cancer | map05200 | 0.002 | 2.824 | 0.031 |
| Inflammatory mediator regulation of TRP channels | map04750 | 0.003 | 2.585 | 0.037 |
| Caprolactam degradation | map00930 | 0.001 | 2.854 | 0.038 |
| Biosynthesis of alkaloids derived from terpenoid and polyketide | map01066 | 0.005 | 2.337 | 0.049 |
| Aldosterone synthesis and secretion | map04925 | 0.001 | 2.921 | 0.049 |
| Bile secretion | map04976 | 0.018 | 1.738 | 0.050 |

**^*^**Comparisons between TC patients and HCs.

TC, thyroid cancer; HC, healthy control; KEGG, Kyoto Encyclopedia of Genes and Genomes;

**Supplemental Table 9 RDA/CCA analysis of fecal flora and 10 lipids compounds**

| **Lipids compounds** | **CCA1** | **CCA2** | ***r^2^*** | ***P* value** |
| --- | --- | --- | --- | --- |
| 27-Hydroxycholesterol | 0.7913 | -0.6115 | 0.3838 | 0.001 |
| Cholesterol | 0.9737 | -0.2279 | 0.2637 | 0.001 |
| 7-Dehydrocholesterol | 0.9862 | 0.1655 | 0.2497 | 0.001 |
| Deoxycholic acid glycine conjugate | 0.9916 | 0.1292 | 0.2168 | 0.001 |
| Ricinoleic acid | -0.8797 | -0.4755 | 0.2065 | 0.001 |
| Linoelaidic Acid | -0.6024 | -0.7982 | 0.1084 | 0.003 |
| Aminocaproic acid | -0.7017 | 0.7125 | 0.0867 | 0.009 |
| DG (16:0/18:0/0:0) | -0.9158 | -0.4017 | 0.0757 | 0.022 |
| Phytosphingosine | 0.9318 | -0.3631 | 0.0716 | 0.027 |
| Solasodine | 0.9948 | 0.1021 | 0.0383 | 0.114 |

CCA, canonical correspondence analysis; DG, diacylglycerol.
